# Prognostic Role of Pretreatment Tumor Burden and Dissemination Features From 2‐[^18^F]FDG PET/CT in Advanced Mantle Cell Lymphoma

**DOI:** 10.1002/hon.70009

**Published:** 2024-11-29

**Authors:** Domenico Albano, Nicola Bianchetti, Anna Talin, Francesco Dondi, Alessandro Re, Alessandra Tucci, Francesco Bertagna

**Affiliations:** ^1^ Nuclear Medicine Department ASST Spedali Civili Brescia Brescia Italy; ^2^ Università degli Studi di Brescia Brescia Italy; ^3^ Division of Hematology ASST Spedali Civili Brescia Italy

**Keywords:** 18F‐FDG PET/CT, dissemination, Dmax, Mantle cell lymphoma, MTV, prognosis

## Abstract

Mantle cell lymphoma (MCL) is an aggressive non‐Hodgkin lymphoma with poor prognosis. The usefulness of fluorine‐18‐fluorodeoxyglucose positron emission tomography/computed tomography (2‐[^18^F]FDG PET/CT) and its parameters in the evaluation of treatment response and prognosis is not yet clear. The aim of this study was to investigate the prognostic role of tumor burden and tumor dissemination features derived by 2‐[^18^F]FDG PET/CT in advanced MCL. We retrospectively included 120 patients with advanced MCL who underwent baseline 2‐ 2‐[^18^F]FDG PET/CT and end‐of‐treatment (eot) PET/CT. The baseline‐PET images were analyzed visually and semi‐quantitatively by measuring the maximum standardized uptake value body weight (SUVbw), lean body mass (SUVlbm), body surface area (SUVbsa), metabolic tumor volume (MTV), total lesion glycolysis (TLG) and dissemination features (Dmax and Dmax‐bsa). EotPET/CT was judged according to the Lugano classification. Progression‐free survival (PFS) and overall survival (OS) were plotted according to the Kaplan–Meier method. At a median follow‐up of 59 months, relapse/progression occurred in 68 patients while death in 38 patients with a median PFS and OS of 27.2 and 57.6 months, respectively. MIPI score, Bulky disease, Ki‐67 index, metabolic response, pretreatment MTV and TLG were significantly associated with PFS at univariate analysis, but only metabolic response, MTV and TLG were confirmed to be independent prognostic factors. Considering OS, only dissemination features were demonstrated to be prognostic features. In conclusions, metabolic response and metabolic tumor burden parameters (MTV and TLG) are strongest predictor of PFS, while dissemination features may have a significant role for predicting OS.

## 1 Introduction

Mantle cell lymphoma (MCL) is a rare subtype of aggressive B cell non‐Hodgkin's lymphoma (NHL) representing about 3%–10% of all NHLs in Western countries [1]. The median age of diagnosis is between 60 and 70 years, while clinical presentations are quite various (ranging from asymptomatic indolent clinical course to symptomatic appearance). The pathogenesis of MCL is very complex including several molecular aberrations, environmental risk factors, and/or familiar risk [2]. MCL usually has a high risk of relapse and poor prognosis despite recent improvements in the treatment field [3]. At the moment, several biological, pathological, and imaging markers to stratify these patients are studied with controversial results. A specific prognostic index for MCL was created and called The Mantle Cell Lymphoma International Prognostic Index (MIPI) [4] and demonstrated to be a strong predictor of outcome. However, also other prognostic markers, such as blastoid variant, Ki‐67 index higher than 30%, and TP53 mutation/deletion were tested with positive findings [5, 6, 7]. Preliminary evidence about a potential prognostic role of fluorine‐18‐fluorodeoxyglucose positron emission tomography/computed tomography (2‐[^18^F]FDG PET/CT) in MCL has been emerging, especially concerning metabolic tumor burden features and metabolic response after treatment applying Lugano criteria [8, 9, 10]. Lugano treatment response classification is a system based on the application of the Deauville five‐point scale for reporting response by 2‐[^18^F]FDG PET/CT in HL and several NHL [11]. Recently, a new PET‐derived metabolic variable describing the dissemination of the hypermetabolic disease in the body was studied in lymphomas: the maximum tumor dissemination (Dmax) [12]. Dmax was defined as the maximum distance between the two farthest lesions with increased uptake on 2‐[^18^F]FDG PET/CT. In MCL, only one research investigated the role of Dmax showing no prognostic usefulness of this feature [10]. A more sophisticated prognostic stratification model is desirable to identify subgroups who might benefit from more aggressive therapies, or in whom the prognosis is already sufficiently good to obviate more conservative treatment plans. For this reason, the idea to incorporate in this prognostic model also metabolic features may be of clinical interest. This study aimed to analyze whether 2‐[^18^F]FDG PET/CT and its parameters alone or combined with classical clinical and epidemiological variables may prognosticate outcome in MCL.

## 2 Materials and Methods

### 2.1 Patients Selection

**FIGURE 1:**
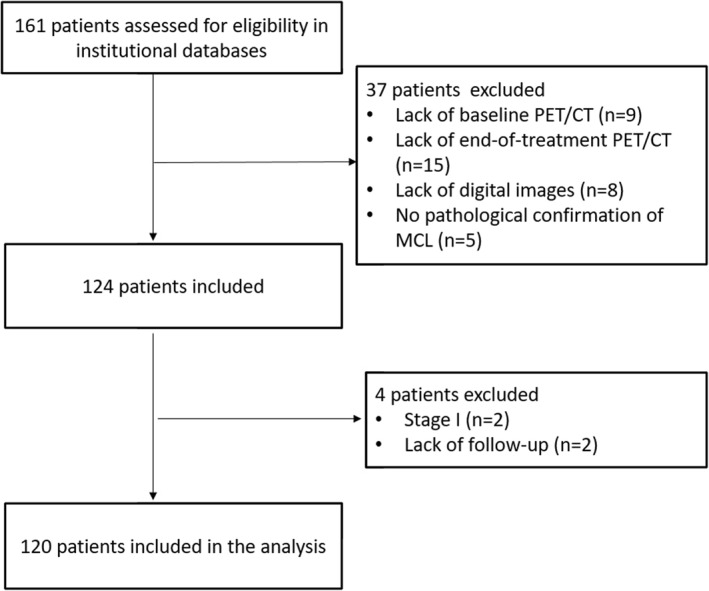
Flow diagram of patients included in the study.

This research was a monocentric retrospective study. Using our institutional Radiology Information System (RIS), we have screened all the patients studied with 2‐[^18^F]FDG PET/CT in our Nuclear Medicine center from February 2007 until January 2023. Inclusion criteria were (1) histological diagnosis of MCL, (2) presence of baseline and end‐of‐treatment (eot) 2‐[^18^F]FDG PET/CT, (3) intermediate‐advanced stage disease (stage II, III, IV), (4) absence of concomitant malignancy, (4) presence of at least 12 months of follow‐up. Applying these criteria (Figure 1), 120 patients were included in this research. For each patient, the main epidemiological (sex, age at diagnosis), clinical (MIPI score, Ann Arbor stage, MCL variant, presence of B symptoms, presence of bulky disease, LDH and β2‐microglobulin level at diagnosis, Ki‐67 level, kind of therapy) were collected. To define bulky disease, we considered any mass measuring at least 10 cm or more in diameter by any imaging study, or with a diameter equal or greater than one‐third of the internal transverse diameter of the thorax. Proliferative activity, measured by Ki‐67 score, was available in 106 patients; the Ki‐67 expression level was divided into two groups: ≤ 30% and > 30% as suggested previously in the literature [6]. All patients were treated according to the institution's standard protocol with chemotherapy regimen. Fifty‐eight patients according to R‐CHOP (Rituximab, Cyclophosphamide, Hydroxydoxorubicine, Oncovin and Prednisone) or alternating R‐CHOP/R‐DHAP (Rituximab, Dexamethasone, high dose Ara‐C cytarabine, Cisplatin) regimen followed by autologous stem cell transplantation; 46 patients were treated according to R‐BAC regimen up to six cycles of immuno‐chemotherapy including Rituximab, Bendamustine and Cytarabine; 4 patients received R‐HyperCVAD and the remaining 12 patients were treated according to MCL 0208 trial which consisted of high‐dose chemotherapy additional with Rituximab, followed by autologous stem cell transplantation and Lenalidomide as maintenance therapy. Globally 70 patients received autologous stem cell transplantation. Also elderly patients were treated because in fit status.

### 2.2 2‐[^18^F]FDG PET/CT Imaging and Analysis

Baseline 2‐[^18^F]FDG PET/CT was performed before any kind of treatment (chemotherapy and/or radiotherapy), eotPET/CT was performed after 6 cycles of chemotherapy or less in case of conditions that contraindicated further cycles. Median time from PET/CT and starting treatment was 7 days (range 1–14 days). No patients received a watch and wait approach.

2‐[^18^F]FDG PET/CT was performed after at least 4 h fasting and with glucose blood level less than 150 mg/dL. Sixty minutes after the injection by vein of an activity of 3.5–4.5 MBq/Kg of radiotracer, 2‐[^18^F]FDG PET/CT scans were acquired. Usually, the field of view was from the skull basis to the mid‐thigh. Tomographs where scans were acquired: a Discovery 690 or a Discovery ST scanner (General Electric Healthcare, Milwaukee, WI, USA) with standard CT parameters (80 mA, 120 Kv, without contrast; 2.5–3.5 min per bed‐PET‐step of 15 cm); the reconstruction was performed in a 128 × 128 matrix and 60 cm field of view. Reconstruction protocol consists of ordered subset expectation maximization (OSEM), filter cut‐off 5 mm, 21 subsets and 2 iterations for both scanners.

2‐[^18^F]FDG PET/CT scans for all patients were visually and semiquantitatively revised by an expert nuclear medicine physician (DA) who was blinded to the patient clinical data and outcome.

The PET images were analyzed from a qualitative and semiquantitative point of view. Concerning qualitative analysis, every focal uptake different from physiological distribution and background was considered suggestive of disease. Bone marrow disease was considered if there was a focal uptake; spleen disease was considered if there was focal uptake in the spleen or diffuse uptake higher than 1.5 of the liver background. EotPET were classified according to Lugano criteria applying Deauville Scores [11]. According to Deauville score 2‐[^18^F]FDG PET/CT was interpreted as follows: 1 = no uptake above background, 2 = uptake equal to or lower than mediastinum, 3 = uptake higher than mediastinum, and lower than liver, 4 = uptake moderately increased compared to the liver and 5 = uptake markedly increased compared to the liver. Concerning the Deauville scores, PET/CT scans were defined as a complete metabolic response in the presence of Deauville scores 1–3 and not complete metabolic response in the presence of Deauville scores 4–5. Concerning semiquantitative measurements, we measured the maximum standardized uptake value corrected for body weight (SUVbw), SUV corrected for body surface area (SUVbsa), SUV corrected for lean body mass (SUVlbm), metabolic tumor volume (MTV), total lesion glycolysis (TLG), Dmax and Dmax corrected for bsa. For these measurements, we used LIFEx software [13].

SUV values were measured in the lesion with the highest uptake by drawing a region of interest over the area of maximum activity and the SUVmax was calculated as the highest SUV of the pixels within the ROI. MTV was calculated using the 41% SUVmax threshold as suggested by the European Association of Nuclear Medicine [14]. TLG was subsequently calculated as the sum of MTV*SUVmean for each uptake. To calculate Dmax, the Euclidean formula measured the distance between all pairs of lesions (including both nodal and extranodal) recording the greatest lesion distance. For Dmax‐bsa, we applied the Du Bois method [15].

### 2.3 Statistical Analysis

The statistical analyses were performed with Statistical Package for Social Science (SPSS) version 24.0 for Windows (IBM, Chicago, Illinois, USA). The descriptive analysis of categorical variables included the simple and relative frequencies; the numeric variables as average, standard deviation, minimum and maximum.

Receiver operating characteristic (ROC) curve analyses were used to identify the best thresholds of metabolic parameters in the light of which interpret the results of progression free survival (PFS) and overall survival (OS) (Table S1). PFS was calculated from the date of baseline 2‐[^18^F]FDG PET/CT to the date of first relapse, disease progression, or the date of last follow‐up. Progression/relapse was considered when a dimensional and/or numerical increase of lesion at CT or PET/CT was demonstrated. OS was calculated from the date of baseline 2‐[^18^F]FDG PET/CT to the date of death from any cause or to the date of last follow‐up. Survival curves were plotted according to the Kaplan–Meier method and differences between groups were analyzed by using a two‐tailed log‐rank test. Cox regression was used to estimate the hazard ratio (HR) and its confidence interval (CI). A *p‐value* of < 0.05 was considered statistically significant.

## 3 Results

### 3.1 Tumors Characteristics at Baseline

In our population, there was a prevalence of males (*n* = 90) and the average age was 65.6 years (range 30–89). Most patients were classified as advanced stage (stage IV) with 104 cases, followed by stage III with 11 cases and stage II with 5 cases. Bulky disease, splenomegaly and B symptoms were described in 16, 49 and 29 patients, respectively. MIPI score was low in 35 cases, intermediate in 45 and high in the remaining 39. Ki‐67 index was low (< 30%) in most patients (*n* = 68). 2‐[^18^F]FDG PET/CT resulted all positive showing at least two hypermetabolic lesions. The mean SUVbw of the lesion with higher ^18^F‐FDG uptake at the baseline was 10.4 (range 3.5–14.5); mean SUVlbm 6.9 (range 2.5–9.5), mean SUVbsa 2.5 (range 0.7–6.8), mean MTV 491 cm^3^ (2–4000 cm^3^) mean TLG 3060 (6–23000), mean Dmax 54.6 cm (6–82) and mean Dmax‐bsa 29.5 (3.3–49). All patients had ^18^F‐FDG‐avid nodal disease; extranodal involvement included bone marrow in 34 (28%) patients and gastrointestinal system in 16 (13%). All the main features of the patients are summarized in Table 1.

**TABLE 1:** The main clinical characteristics of the entire population included (*n* = 120 patients).

	Patients *n* (%)	Average ± SD (range)	Median
Sex male	90 (75%)		
Sex female	30 (25%)		
Age (years)		65.6 ± 10 (30–89)	63
Tumor stage at diagnosis (Ann Arbor)			
I	0 (0%)		
II	5 (4%)		
III	11 (9%)		
IV	104 (87%)		
Blastoid variant	16 (13%)		
B symptoms	29 (24%)		
LDH			
Normal	77 (64%)		
Increased	43 (36%)		
β2 microglobulin			
Normal	82 (68%)		
Increased	38 (32%)		
Bulky disease	16 (13%)		
Splenomegaly	49 (41%)		
Ki‐67 score^a^			
< 30%	68 (64%)		
≥ 30%	38 (36%)		
MIPI score			
Low	36 (30%)		
Intermediate	45 (37.5%)		
High	39 (32.5%)		
SUVbw		10.4 ± 6 (3.5–9.7)	9
SUVlbm		6.9 ± 4.1 (2.5–9.5)	6
SUVbsa		2.5–1.5 (0.7–6.8)	2.5
MTV		491 ± 696 (2–4000)	358
TLG		3060 ± 4625 (6–23000)	2950
Dmax		54.6 ± 19.5 (6–82)	48.8
Dmax‐bsa		29.5 ± 11.1 (3.3–49)	25.9

Abbreviations: LDH, lactate dehydrogenase; MIPI, Mantle international prognostic index; MTV, metabolic tumor volume; SUVbsa, body surface area; SUVbw, standardized uptake value body weight; SUVlbm, lean body mass; TLG, total lesion glycolysis.

^a^ Not available in 14 patients.

### 3.2 Treatment Response Setting

Based on Lugano classification, complete metabolic response was registered in 83 (70%) patients, and not complete response in 35 (30%) patients. Two patients died before the execution of eotPET/CT. Among complete metabolic responses, Deauville scores 1,2,3 were defined in 62, 12 and 9 patients, respectively. Among not complete metabolic responses, Deauville scores 4 and 5 were present in 19 and 16 cases. Comparing complete and not complete response groups, only age and Ki‐67 scores were significantly different (*p* = 0.011); particularly, age and Ki‐67 index were significantly higher in patients that did not reach complete response. No significant difference was demonstrated comparing the metabolic PET features between not complete and complete response groups (Table 2).

**TABLE 2:** Comparison of baseline metabolic PET/CT features between no response and complete/partial response groups at end‐of‐treatment.

Parameter	End of treatment response		
	Complete metabolic response *n* 83	Not complete metabolic response *n* 35	*p* value
Gender M: F	61:22	29:8	0.680
Age, mean ± SD	64.1 ± 12	69 ± 13	0.011
Stage IV	71 (68%)	33 (94%)	0.553
Blastoid variant	9 (11%)	7 (20%)	0.578
B symptoms	20 (24%)	9 (26%)	0.685
LDH increased	25 (30%)	18 (51%)	0.787
β2 microglobulin increased	22 (27%)	16 (46%)	0.459
Bulky disease	11 (13%)	5 (14%)	0.882
Splenomegaly	35 (42%)	14 (40%)	0.828
Ki‐67 score ≥ 30%	21 (25%)	17 (49%)	0.011
MIPI score intermediate/high	56 (67%)	28 (80%)	0.172
SUVbw, mean ± SD	8.7 ± 3.1	9.7 ± 4	0.320
SUVlbm, mean ± SD	6.5 ± 2.7	7.3 ± 2.8	0.347
SUVbsa, mean ± SD	2.2 ± 0.8	2.5 ± 0.9	0.195
MTV, mean ± SD	486 ± 99	530 ± 121	0.757
TLG, mean ± SD	2810 ± 1450	3831 ± 1345	0.283
Dmax, mean ± SD	55.5 ± 19.8	54 ± 21.2	0.718
Dmax bsa, mean ± SD	29.9 ± 10.1	29.4 ± 12	0.835

Abbreviations: bsa, body surface area; bw, body weight; F, female; lbm, lean body mass; M, male; MIPI, Mantle Cell Lymphoma International Prognostic Index; MTV, total metabolic tumor volume; SD, standard deviation; SUV, standardized uptake value; TLG, total lesion glycolysis.

### 3.3 Role of 2‐[^18^F]FDG PET/CT in Predicting PFS

**FIGURE 2:**
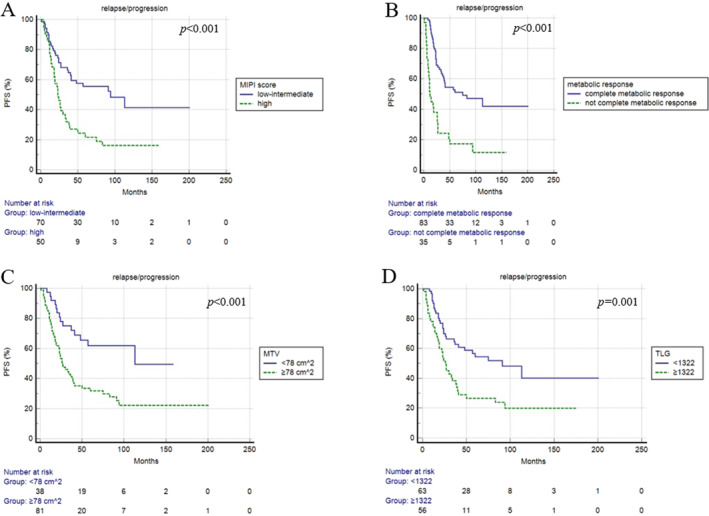
Progression free survival curves according to MIPI score (A), metabolic response (B), MTV (C) and TLG (D).

**FIGURE 3:**
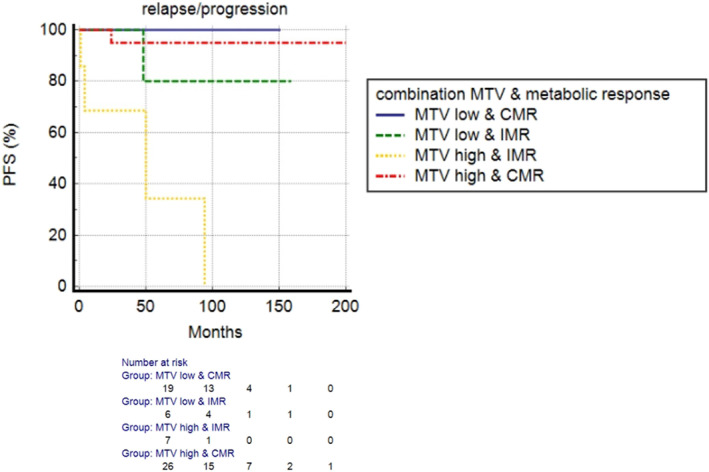
Combination of baseline MTV and metabolic response to predict PFS.

At a median follow‐up of 59 months, relapse or progression of disease occurred in 68 patients with an average time of 26 months (range: 2–113 months) from the baseline PET/CT. Median PFS was 27.2 months. Three‐year and 5‐year PFS were 50% and 36%, respectively. At univariate analysis, the presence of bulky disease, Ki‐67 level, MIPI score, metabolic response at eotPET/CT, MTV and TLG were significantly associated with PFS (Table 3). PFS was statistically significantly longer in patients with low‐to‐intermediate MIPI score, complete metabolic response at eotPET/CT DC, low MTV and low TLG (Figure 2). At multivariate analysis, eotPET/CT, MTV and TLG were confirmed to be independent prognostic factors (*p* < 0.001; *p* = 0.003; *p* = 0.042) (Table 4). Concerning PFS, dissemination features were not associated with the outcome. The combination of metabolic response and baseline MTV stratified better patients PFS (Figure 3). Patients with high MTV and incomplete response at eotPET/CT had the worse PFS. The median PFS of patients with low MTV and complete metabolic response was 58.9 months, of the patients with low MTV and incomplete metabolic response 48 months, of the patients with complete response and high MTV 29.7 months and of patients with incomplete metabolic response and high MTV 8 months.

**TABLE 3:** Univariate analyses for progression free survival and overall survival.

	PFS		OS	
	*p* value	HR (95% CI)	*p* value	HR (95% CI)
Sex	0.483	1.600 (0.586–3.001)	0.610	1.135 (0.705–1.825)
Age	0.353	1.700 (0.666–4.123)	0.522	1.167 (0.736–1.853)
Stage IV	0.765	1.106 (0.569–2.147)	0.070	2.253 (0.607–8.369)
B symptoms	0.320	1.528 (0.652–2.321)	0.218	1.363 (0.795–2.339)
Blastoid variant	0.415	0.850 (0.528–1.859)	0.650	0.863 (0.472–1.578)
LDH increased	0.405	0.444 (0.650–2.006)	0.390	0.609 (0.450–1.890)
β2 microglobulin increased	0.601	2.021 (0.555–4.002)	0.777	2.021 (0.555–4.002)
Bulky disease	0.030	2.449 (1.093–5.503)	0.301	0.745 (0.445–1.245)
Splenomegaly	0.858	1.045 (0.640–1.706)	0.266	0.778 (0.503–1.202)
Ki‐67 score high	0.014	2.079 (1.156–3.740)	0.761	1.079 (0.655–1.775)
MIPI score high	< 0.001	2.626 (1.574–4.384)	0.596	1.130 (0.712–1.793)
Metabolic response	< 0.001	5.184 (2.727–9.852)	0.917	1.028 (0.599–1.768)
SUVbw^a^	0.685	1.105 (0.681–1.792)	0.489	1.165 (0.751–1.809)
SUVlbm^a^	0.351	1.261 (0.774–2.055)	0.802	1.057 (0.685–1.631)
SUVbsa^a^	0.218	1.360 (0.833–2.221)	0.465	1.176 (0.762–1.185)
MTV^a^	< 0.001	2.344 (1.431–3.839)	0.308	0.797 (0.509–1.247)
TLG^a^	0.001	2.220 (1.348–3.655)	0.338	0.800 (0.514–1.247)
Dmax^a^	0.304	1.299 (0.788–2.143)	0.039	1.590 (1.031–2.452)
Dmax bsa^a^	0.106	1.489 (0.917–2.416)	0.037	1.587 (1.00–2.467)

Abbreviations: bsa, body surface area; bw, body weight; CI, confidence interval; HR, hazard ratio; lbm, lean body mass; MTV, total metabolic tumor volume; *N*°, number; OS, overall survival; PFS, progression free survival; SUV, standard uptake value; TLG, total lesion glycolysis.

^a^ Dichotomized according to ROC analysis.

**TABLE 4:** Multivariate analyses for progression free survival and overall survival.

	PFS		OS	
	*p* value	HR (95% CI)	*p* value	HR (95% CI)
Stage IV			0.818	0.905 (0.389–1.205)
Bulky disease	0.056	2.143 (0.998–4.550)		
Ki‐67 score high	0.878	1.047 (0.580–1.890)		
MIPI score high	0.074	1.366 (0.970–1.924)		
Metabolic response	< 0.001	3.462 (1.887–6.349)		
MTV^a^	0.003	2.732 (1.387–5.382)		
TLG^a^	0.042	1.814 (1.019–3.229)		
Dmax^a^			0.134	1.120 (0.890–1.409)
Dmax bsa^a^			0.025	1.745 (1.070–2.844)

Abbreviations: CI, confidence interval; HR, hazard ratio; MTV, total metabolic tumor volume; OS, overall survival; PFS, progression free survival; TLG, total lesion glycolysis.

^a^ Dichotomized according to ROC analysis.

### 3.4 Role of 2‐[^18^F]FDG PET/CT in Predicting OS

**FIGURE 4:**
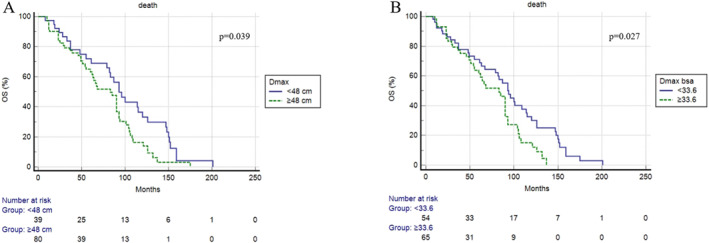
Overall survival curves according to baseline Dmax (A) and Dmax‐bsa (B).

Death occurred in 38 patients with an average time of 37 months (range 2–151). In most patients (*n* = 27) the death was considered directly related to the lymphoma or therapy; in the remaining 11 cases the causes of death are not related to the lymphoma. The median OS was 57.6 months. Three‐year and 5‐year OS were 78% and 65%, respectively. At univariate analysis, stage IV, Dmax and Dmax‐bsa were significantly correlated with OS (Table 3). OS was statistically significantly longer in patients with low Dmax and low Dmax‐bsa (Figure 4). At multivariate analysis, only Dmax bsa was confirmed to be an independent prognostic variable (*p* = 0.025) (Table 4). Concerning OS, all the other metabolic features showed to have no prognostic value.

## 4 Discussion

MCL demonstrated to be a lymphoma variant with a high 2‐[^18^F]FDG PET/CT detection rate concerning nodal diseases, while the diagnostic performances of PET/CT in the detection of gastrointestinal involvement and bone marrow (BM) infiltration are less good [16]. This is the reason why it is suggested to perform BM biopsy and GI endoscopy in the staging phase to classify correctly these patients. Both BM and the gastrointestinal tract are hard settings to examine with ^18^F‐FDG, due to the presence of physiologic uptake of this radiopharmaceutical in several inflammatory/infectious and functional conditions.

On the other hand, in the restaging field, especially in the evaluation of treatment response, 2‐[^18^F]FDG PET/CT seems to be an accurate tool better than morphological examinations [16]. Another potential advantage of PET/CT is the possibility to give prognostic information to predict disease aggressiveness and consequently affect patient management.

The first result of this study was the validation of the prognostic role of metabolic response categories applying Deauville scores. Deauville scores is a scale using the liver and blood‐pool activity as the references to classify the degree of uptake of residual disease after therapy and it has been recommended for reporting both interim and end‐of‐treatment PET for HL and several NHL, like FL and DLBCL [11]. Also in MCL setting, this scale seems to have a strong impact [17, 18].

In our study we demonstrated that patients with a complete metabolic response after first‐line therapy had significantly longer PFS than patients with incomplete response; but this finding was not confirmed for OS.

In addition to qualitative analysis, also semiquantitative baseline metabolic parameters were studied for prognostic purposes. Previous studies focused on the potential prognostic role of baseline SUV with controversial results [9, 19, 20, 21, 22]. However, also in the research [20, 22] where SUVmax was demonstrated to be a good predictor of outcome the thresholds suggested were very different (i.e. 5 and 10.3). In our analysis, no SUV‐related parameters (corrected for body weight, for lean body mass, for body surface area) showed to have a prognostic impact. SUV is the most widely utilized and generally accepted parameter in the current published literature because it is a feature very easy to extract, automatic and fast but less reproducible due to the potentially influence of several variables, like uptake time, decay of radiotracer, blood glucose level, risk of extravasation of tracer, lesion size and technical features (scanner‐related, acquisition protocol‐related and reconstruction protocol related).

To overtake these limitations, other metabolic parameters were introduced with success: MTV and TLG. These features expressed intrinsically both morphological/volumetric and metabolic characteristics of the lesions measured and demonstrated to be strong factors in several lymphoma variants [23]. Concerning MCL, positive evidence are available [9, 22] but based on low population sample. Our study confirmed the role of MTV and TLG in the largest cohort size (*n* = 120). Baseline MTV and TLG, which represent a combination of tumor volume and metabolism, were robust predictor of outcome, but only for PFS. Nevertheless, the application of MTV and TLG in clinical routine practice could probably be premature, because of the lack of a standardized method for their measurement. Different methods are proposed and a wide range of threshold levels have been used to measure the volume‐based PET/CT variables. The most common method (used also in this research) utilized an isocontour threshold method based on 41% of the SUVmax, as suggested by the EANM guideline [14]. But also fixed absolute threshold (SUVmax 2.5 or SUVmax 4) or adaptive methods are described in the literature [24].

However, MTV and TLG did not consider in their definition the distribution of hypermetabolic disease and the number of lesions FDG‐avid. MCL usually present with advanced stage, plural nodal localization and frequent extranodal involvement. Dmax is a parameter that represents the tumor distribution and dissemination of disease with increased uptake in the body. The potential advantages of Dmax compared to MTV and TLG are the simplicity, velocity of extraction (now automatic with different software) and clinical explanation. Moreover, Dmax is not directly affected by PET/CT tomograph characteristics or PET protocols like SUV.

However also Dmax is a variable with unexplored potential and question marks. For example, Dmax as absolute value did not consider patient body composition, like height and weight. For this reason, we decided to calculate both Dmax and Dmax corrected for body surface area applying DeBois method. This difference seemed to have an impact in our study, because in the multivariate analysis of OS only Dmax corrected for bsa showed to be an independent prognostic element.

More innovative parameters, such as sarcopenia parameters and radiomics features, are tested in the literature and also in MCL [25, 26].

Larger studies are required to find the best combination of prognostic factors and the factors most readily used in clinical practice for MCL.

Recently, Vergote et al. [10] investigated prognostic role of several PET variables (SUV, MTV, TLG and dissemination features) in untreated MCL and demonstrated that among PET features only MTV was significantly associated with PFS, while dissemination features (Dmax) had no prognostic impact. These findings were partially confirmed in our investigation, where Dmax‐bsa showed to be independent prognostic factor only for OS, not for PFS.

Concerning PFS, we hypothesized a prognostic scoring system based on PET/CT metabolic features derived from the baseline and eotPET/CT scan that can be complementary and describe two different attributes of the disease: tumor burden and metabolic response after treatment. This score might possibly be helpful to predict prognosis after first‐line treatment identifying patients with higher risk of recurrence/relapse. In the clinical practice, this model could anticipate the imaging controls or associate a more aggressive treatment approach after first‐line. Of course, this score needs to be validated in a more robust population and with a longer follow‐up period. However, the combination of both features (high baseline MTV and incomplete metabolic response) is associated with worse PFS as shown in Figure 4.

One of the main finding of this manuscript is the prognostic role of Dmax in OS, but not in PFS. The reason of this discrepancy remains unclear and deserved deeper evaluation.

The potential limitations of our study are the retrospective nature of the study, the long period of inclusion of patients, and the heterogeneity of patients (as different therapeutic regimens and clinical/epidemiological features). Despite this, so far, the present study represents the largest series of MCL investigated with a visual and semiquantitative analysis of 2‐[^18^F]FDG PET/CT and their prognostic role.

## 5 Conclusion

In conclusion, with this study, we demonstrated that metabolic response at eotPET/CT (evaluated according to Deauville criteria) and the baseline metabolic tumor features (MTV and TLG) were significantly correlated with PFS. Instead, only Dmax corrected for‐bsa was correlated with OS.

## Ethics Statement

All procedures performed in studies involving human participants were in accordance with the ethical standards of the institutional and/or national research committee and with the 1964 Helsinki declaration and its later amendments or comparable ethical standards. For this type of study formal consent is not required.

## Consent

Informed consent was obtained from all individual participant included in the study.

## Conflicts of Interest

The authors declare no conflicts of interest.

### Peer Review

The peer review history for this article is available at https://www.webofscience.com/api/gateway/wos/peer-review/10.1002/hon.70009.

## Supporting information

Table S1

## Data Availability

The data that support the findings of this study are available on request from the corresponding author. The data are not publicly available due to privacy or ethical restrictions.
