# Supplementary material for: Prognostic Role of Pretreatment Tumor Burden and Dissemination Features From 2‐[18F]FDG PET/CT in Advanced Mantle Cell Lymphoma
Source: Hematol Oncol. 2024 Nov 29;43(1):e70009. doi: 10.1002/hon.70009 (PMC11607474; doi:10.1002/hon.70009)
Supplement: Supplementary file 1 — Table S1 [file HON-43-e70009-s001.docx]

**Supplemental Table** **1**: Receiver operating characteristic (ROC) curve analysis of semiquantitiave PET/CT features

|  | ROC curve | | | | |
| --- | --- | --- | --- | --- | --- |
| **Parameter** | cutoff | AUC (95% CI) | *p* value | Sensitivity (95% CI) | Specificity (95% CI) |
| SUVbw | 7.7 | 0.590 (0.496-0.679) | 0.108 | 56% (44.7-67) | 62% (44.8-77.5) |
| SUVlbm | 5.2 | 0.588 (0.494-0.678) | 0.122 | 48% (36.4-58.9) | 70% (53-84.1) |
| SUVbsa | 1.7 | 0.579 (0.485-0.669) | 0.156 | 45% (34.1-56.5) | 77% (58.8-88.2) |
| MTV | 78 | 0.683 (0.591-0.765) | <0.001 | 44% (33-55.3) | 92% (78.1-98.3) |
| TLG | 1322 | 0.688 (0.596-0.778) | <0.001 | 67% (55.8-77.1) | 75.6% (58.8-88.2) |
| Dmax | 48 | 0.564 (0.470-0.655) | 0.252 | 39% (28.4-50.4) | 78% (61.8-90.2) |
| Dmax bsa | 33.6 | 0.556 (0.462-0.646) | 0.323 | 54% (42.5-64.7) | 66 (48.6-80.4) |

*AUC:* area under curve; *CI*: confidence interval; *SUV:* standardized uptake value; *bw*:body weight; *lbm:* lean body mass; *bsa:* body surface area; *tMTV:* total metabolic tumor volume; *TLG:* total lesion glycolysis.
